# Stress Distribution in Peri-implant Bone in the Replacement of Molars with One or Two Implants: A Finite Element Analysis

**DOI:** 10.30476/dentjods.2022.92584.1659

**Published:** 2023-03

**Authors:** Asieh Mozaffari, Donya Hashtbaran, Alireza Moghadam, Shima Aalaei

**Affiliations:** 1 Dept. of Periodontics, Faculty of Dentistry, Qazvin University of Medical Sciences, Qazvin, Iran; 2 Student Research Committee, Qazvin University of Medical Sciences, Qazvin, Iran; 3 Dept. of Prosthodontics, Dental Caries Prevention Research Center, Qazvin University of Medical Sciences, Qazvin, Iran

**Keywords:** Single-Tooth Implants, Dental Prosthesis, Implant-supported, Finite Element Analyses, Molar

## Abstract

**Statement of the Problem:**

In most cases, insertion of single implants with a standard diameter is used to replace a molar tooth but placing two implants with a narrow diameter seems to be a viable treatment modality to withstand functional and biomechanical forces.

**Purpose:**

This study aimed to evaluate and compare stress distribution in the bone surrounding a single molar area rehabilitated by a single implant versus two implants with a narrow diameter.

**Materials and Method:**

The study was conducted by computer-aided *in vitro* modeling. The initial model used a single implant, 4.8 mm wide in diameter, inserted with a 3.9-mm distance from both sides and 12.6-mm mesiodistal space. The second model used two 3.3-mm narrow-sized implants with a 3-mm distance from one another, 1.5 mm from both sides, and a 12.6-mm mesiodistal space. Following the completion of these models, a 100-N force was exerted obliquely, once in three locations and once in the mesial aspect of the implant-supported crown. Stress distribution was then measured using finite element analysis (FEA) with ANSYS Workbench software package in both models.

**Results:**

The maximum stress in the bone around the single implant was less than that around double implants. The maximum stress of cortical bone in three-point loading was lower than mesial loading either in one (146.7 vs. 126.72 MPa) or two implants model (186.8 vs. 139.24).

**Conclusion:**

According to the results, because of more cortical bone contact area, the stress of surrounding bone in wide implant was decreased.

## Introduction

The use of implants in full- and partial-mouth reconstructions improves masticatory function, increases patients’ satisfaction, and improves their quality of life. The mandibular first molar is the most frequent tooth replaced by implants [ 1 ]. One or two implants can be used to replace mandibular first molars. In treatment with two implants, the mesiodistal bending is limited, and both implants resist buccolingual forces [ 2 ], the risk of screw loosening decreases, the size of the cantilever reduces [ 3 ], and invasive surgeries are avoided [ 2 ]. However, there is a doubt about the quality of new bone at the graft site [ 4 - 5 ]. Nonetheless, in some cases, graft surgery is impossible [ 6 ]; therefore, one or two standard implants cannot be inserted, and only two narrow-diameter implants can be used. Nevertheless, the stress distribution in peri-implant bone in this treatment plan is not clear. 

In the study of Desai *et al*. [ 7 - 8 ], two-splinted implant was better than single-implant in stress distribution of the bone around the implants, but in the study of de Carvalho [ 3 ], stress distribution in the bone around the single-implant was better than double-implant. Also, in the study by Hotta *et al*. [ 6 ], the survival rate of single implant was similar to double-implant. 

The finite element analysis (FEA) is a numerical method to characterize stress and strain geometry in a three-dimensional system [ 1 ]. It is also used in dentistry to predict the distribution of stress and strain in implant components and peri-implant bone under different conditions [ 2 ]. Since increased stress in the peri-implant bone increases the odds of physiological bone resorption and failure, it is necessary to identify treatment plans that increase stress.

Considering there was a controversy among the previous studies therefore, this study compared stress distribution in peri-implant bone in replacing mandibular first molars with one standard or two narrow implants using FEA. The null hypothesis of this study was defined as in replacing mandibular first molar; the stress distribution of surrounding bone of single- and double-implant was similar.

## Materials and Method

This study was approved by the Ethics Committee of Qazvin University of Medical Sciences under the code IR.QUMS.REC.1395.188.

The current *in vitro* study employed a computer modeling technique in which two models were constructed and compared. In the first model, a bone-level titanium implant (Straumann, Switzerland), measuring 4.8mm in diameter, 10mm in length, and 3.9mm in distance, on each side, was placed in a 12.6-mm mesiodistal space. In the second model, two 3.3-mm titanium bone-level narrow implants (Straumann, Switzerland), with a 3-mm inter-implant distance and 1.5 mm away from each side, were placed in a 12.6-mm mesiodistal space, too. The implants and their prosthetic components were geometrically designed using Straumann Catalog, the manufacturer’s information and reverse engineering by a computer (Intel® Xeon® CPUE5410 @ 2.33 GHz 8 GB Ram).

**Figure 1:**
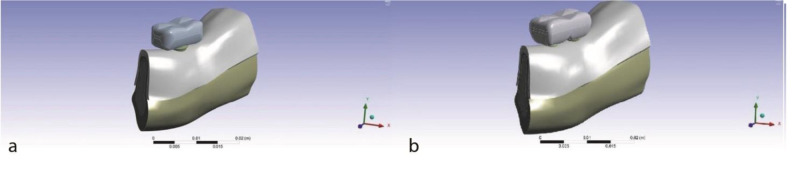
The two treatment plan models, **a:** One implant, **b:** Two splinted narrow-diameter implants

The micro computed tomography (micro-CT) scan image files of a patients’ mandible were used for mandible reconstruction. Then this file was imported into Mimics software version 19 to obtain the actual size and site of cortical and cancellous bone, soft tissues, and the left first molar site and shape. The first molar form was used to reconstruct the shape of the metal-ceramic restorations (MCR) crown. Based on the details of the mandible, in the Mimics software file, the mandible was modeled in the Solidwork 2019 environment, and the teeth were removed from the model. The fixture abutment model was assembled and inserted in the first molar area in the virtual model. The fabricated MCR crown was replaced on the abutment. To create the same conditions in both models, a similar molar crown was constructed for the two models (Figure 1). Then, the data were imported into ANSYS Workbench software version 2019 for analysis. 

A 100-N static oblique force with a 45° to the vertical line was applied only once overall occlusal surface at three points: mesial, central, and distal, only once at the mesial point on the crown. The lower part of the mandible was considered an anchor, and the condyle was assumed virtually. Complete osseointegration was considered in the current study. Between all the other components’ surfaces, close contacts were considered and a frictional bond was designed. 

All the materials used in this study were considered isotropic, homogeneous, and linear elastic. The mechanical properties of the material were obtained from previous literature (Table 1) [ 9 ].

**Table 1:** Mechanical properties of the studied materials

Material	Young’s modulus (Mpa)	Poisson’s ratio
Cortical bone	13700	0.30
Spongy bone	1370	0.30
Feldspathic Porcelain	82800	0.28
NiCr alloy	206000	0.30
Titanium	110000	0.33

**Figure 2:**
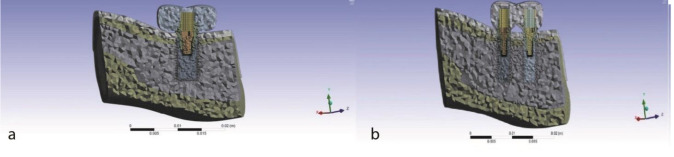
The meshed model in the two treatment plans, **a:** One implant, **b:** Two splinted narrow-diameter implants

The complete 3D models were meshed using the pyramidal elements measuring 30–800 μm (Figure 2).

Finally, the single-implant model had 640619 elements and 1068458 nodes, and the two-implant model had 617679 pyramidal elements and 1021624 nodes. The stresses in the peri-implant bone were determined using the von Mises model [ 7 ].

## Results

### Application of a 100-N force at three points

The maximum von Mises stress values in two- and one-implant models were 139.24 and 129.72 MPa, respectively. In the two-implant model, it was approximately 10 MPa higher than the one-implant model.

**Figure 3:**
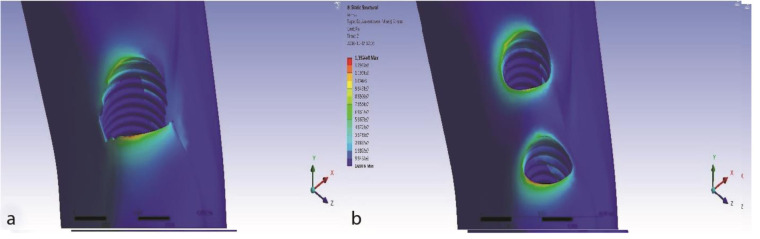
The von Mises diagram of cortical bone for two treatment plans at three-point loading (isometric view), **a:** One implant, **b:** two splinted narrow-diameter implants

Maximum stress was observed in the cervical cortical bone. The maximum stress in the bone around the one-implant model was on the mesial side and in the two-implant model on the mesial side of the mesial implant. The extent of maximum von Mises stress in the bone around the mesial implant of the two-implant models was similar to the single implant (Figure 3). 

### Application of a 100-N mesial force at one point

**Figure 4:**
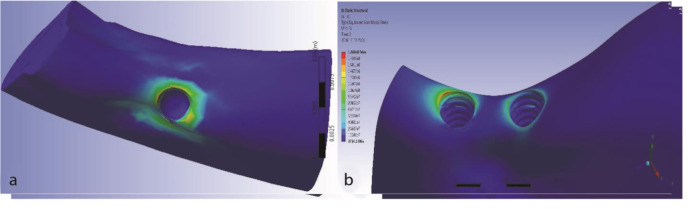
The von Mises diagram for two treatment plans at mesial loading (isometric view), **a:** One implant, **b:** Two splinted narrow-diameter implants

The maximum von Mises stress values in the two- and one-implant designs were 186.8 and 146.7 MPa, respectively. The maximum stress value in the two-implant model was 65 MPa higher than the one-implant model, and the maximum stress was observed in the cortical bone. In the two-implant design, the maximum stress was observed in the mesial bone of the mesial implant and in the one-implant model in the mesial bone of a single implant. The extent of maximum stress in the peri-implant bone was similar in both models (Figure 4).

## Discussion

The null hypothesis of this study was rejected because the stress distribution of surrounding bone of double-implant was not similar to single-implant.

The long-term success of implants depends on stress distribution in the surrounding bone [ 10 ]. The present study compared bone stress distribution in replacing a molar tooth with one or two implants using FEA. 

In mesial loading, the stress of bone on the mesial aspect of the mesial implant in the two-implant model and mesial bone of the single implant in the one-implant model was higher than the other aspect due to the proximity to the site of force applied.

Maximum von Mises stress of cortical bone in three-point loading was lower than mesial loading in either one (146.7 vs. 126.72 MPa) or two implants model (186.8 vs. 139.24) because of the higher concentration of the stress in one-point loading compared to surface loading. The reduction of stress in three-point loading in the two narrow implants was more significant than the single implant (47 Mpa vs. 20 Mpa), and the stress was distributed approximately equally in the peri-implant bone of two implants due to the removal of the anchor and applying forces to both the mesial and distal implants evenly. 

The von Mises maximum stress value in cortical bone was significantly higher in the two-implant model than the single-implant one in both loading states, and the maximum stress value in the bone surrounding the mesial implant was higher than the distal one and the single implant.

Like the present study, Desai *et al*. in two studies [ 7 - 8 ] used the FEA method to evaluate stresses in the bone around one wide implant and two implants in replacing mandibular molar. In contrast to the present study, in both studies, the Von Mises stress in the bone around a single implant was more than two implants. In addition, in a study by Gerami *et al*. [ 11 ], using FEA, the stress of peri-implant bone in the two-implant model was less than that around the single-implant model. In the present study in the single-implant design, due to the large diameter of the implant and the specific shape of the reconstructed mandible (the buccal depression area), the contact area with the cortical bone extended along the buccal surface of the fixture and was more widespread than in two narrow implants. Nevertheless, in the two-implant design, the implants contacted the cortical bone only in the fixture’s cervical area. Since the differences between cortical bone and titanium’s elastic moduli were less than the cancellous bone, the stresses induced by a single implant to surrounding bone were lower. 

Hotta *et al*. [ 6 ] successfully replaced one maxillary molar with two narrow implants inserted diagonally. They concluded that the positioning of the implants in the cortical bone of buccal or lingual areas was a reason for the success of this treatment plan, which reduced stresses in the bone around the fixture. It is similar to the reason for reduced bone stresses in this study. In the studies by Lemos *et al*. [ 12 ] and Didier *et al*. [ 13 ], the area of cortical bone, that contacting implant was one of the most important factors in the reduced stress level of peri-implant bone.

The study of de Carvalho *et al*. [ 3 ], investigated the stress level in the peri-implant bone of mandibular molars with different planning options. In their research, the polyoxymethylene (POM) model was constructed to simulate an edentulous mandible, and implants were inserted in the model. The highest stress level in all the options was observed in the bone in the mesial area of the implants, and the stress value in the peri-implant bone of two splinted narrow implants model was higher than a single implant that inserted in the mesial or distal areas of ​​the edentulous region. Although the modeling method used in their study was different from that of the current study, the results were similar. In their study, like the current study, the peri-implant bone stress level in a single implant was less than a double implant. However, they constructed mandibles with different materials from those used in this study; therefore, comparison is not possible.

In most clinical studies, replacing a molar tooth with two splinted narrow implants was successful; however, most of these studies have not compared the success rates of these two treatment plans [ 2 , 6 , 14 - 16 ]. 

In a study by Wolfinger *et al*. [ 17 ] on 125 patients, two splinted narrow implants were used for single molar replacement in each case. At a three-year follow-up, 115 patients had two implants, but in ten patients, one implant failed, and the failed implants were not replaced. All the single implants survived during the follow-up and one narrow implant on one side of the edentulous area survived and served as two splinted implants. It shows that despite they have a narrow diameter with a long cantilever, the peri-implant bone stress level was lower than that can be failed them. 

This study’s results were achieved using FEA; despite its extensive application in dentistry, it has some limitations. For instance, the bone was considered homogeneous, linearly elastic, and isotropic, 100% osseointegration was assumed; occlusal forces were considered static, and the mandibular flexure was ignored. 

On the other hand, the exact simulation of clinical conditions is not possible in FEA studies, and the findings of such studies must be used with caution. For example, using two narrow splinted implants was successful in clinical studies [ 2 , 6 , 14 - 16 ] because a higher bone volume around narrow fixtures provides more perfusion and nutrition, increasing the possibility of osseointegration, which cannot be simulated in the FEA model. 

## Conclusion

Given the limitations of this study, in the surrounding bone of the single standard implant, the stress value was less than two splinted narrow implants, because of the unique edentulous ridge shape in the current study that increased contact area with cortical bone in the single-implant model compared with two narrow splinted implants. Therefore, this study showed that the cortical bone-implant contact surface was more important than the total bone-implant contact surface in decreasing peri-implant bone stress level.

## Acknowledgments

The authors of this study would like to thank the staff of the department of prosthodontics, School of Dentistry for their valuable contribution.

## Conflict of Interest

The authors declare that they have no conflict of interest.
